# Supplementary material for: Defining the therapeutic selective dependencies for distinct subtypes of PI3K pathway-altered prostate cancers
Source: Nat Commun. 2021 Aug 20;12:5053. doi: 10.1038/s41467-021-25341-9 (PMC8379232; doi:10.1038/s41467-021-25341-9)
Supplement: Supplementary file 1 — Supplementary Information [file 41467_2021_25341_MOESM1_ESM.pdf]

SUPPLEMENTARY MATERIALS

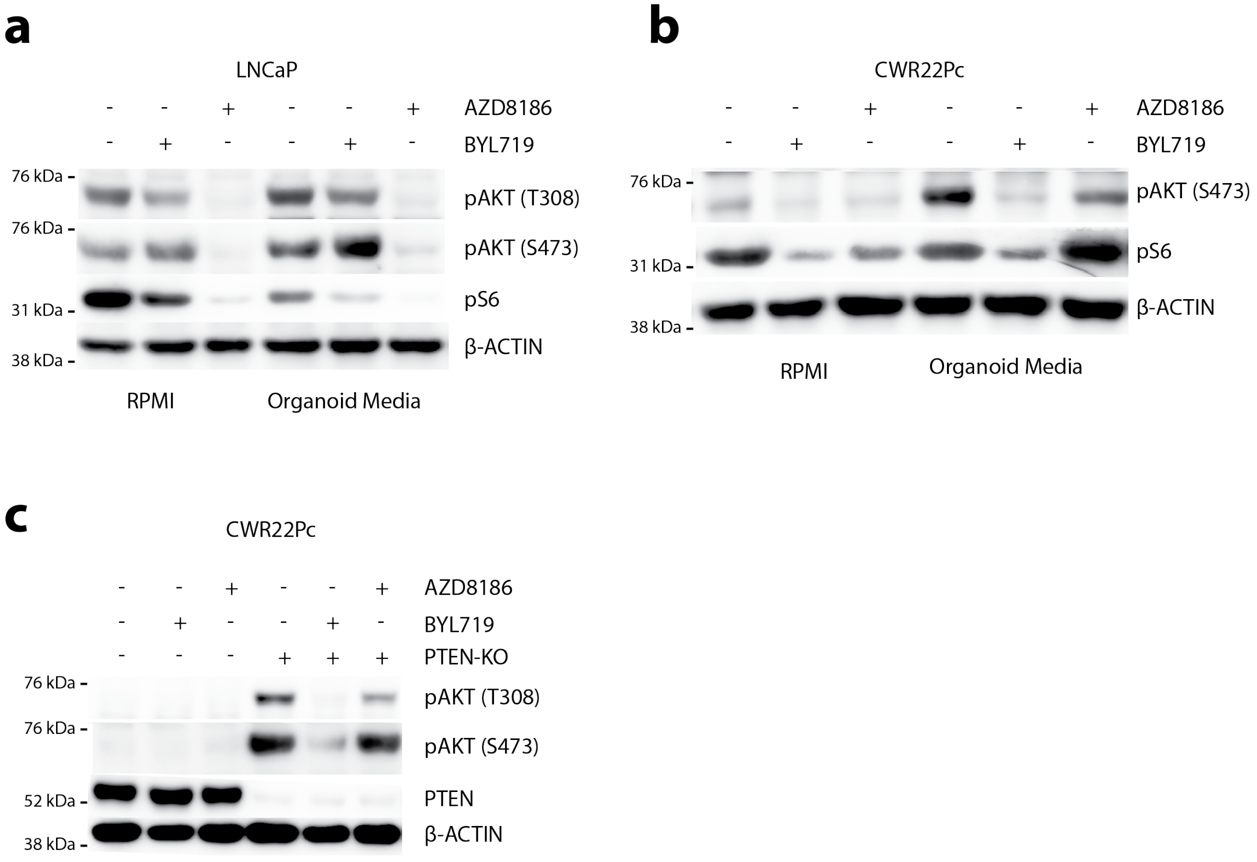

Fig. S1 Modeling PI3K alterations using patient-derived organoid (PDO) reveals PI3K isoform dependency is not determined by *PTEN*

(A, B) Western blot showing levels of pAKT and pS6 in LNCaP or CWR22Pc cells cultured in regular 10% FBS-RPMI or organoid media treated with BYL719 (1μM), AZD8186 (250nM), or vehicle for 4hrs. (C) Western blot showing levels of pAKT in CWR22Pc-sg*NT* or CWR22Pc-sg*PTEN* isogenic pairs treated with BYL719 (1μM), AZD8186 (250nM), or vehicle for 4hrs. Source data are provided as Source Data file. All assays were performed with three biological replicates.

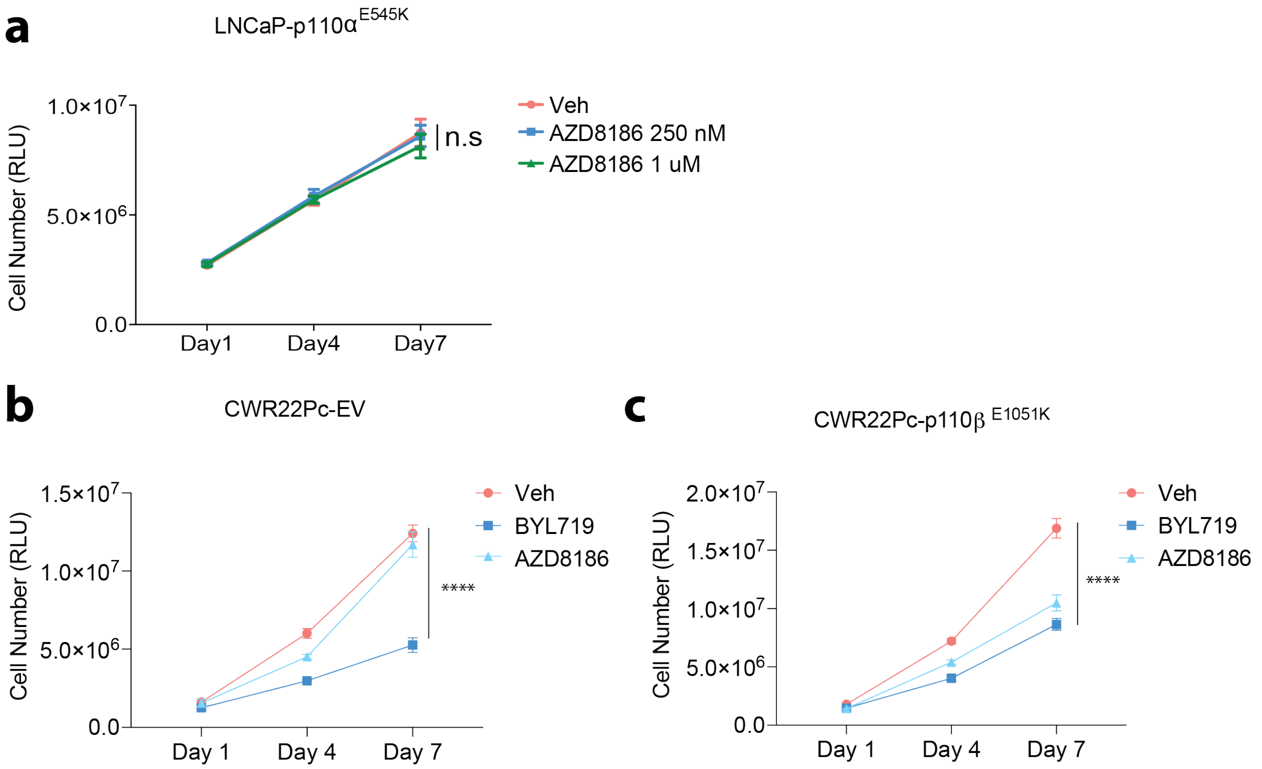

Fig. S2 Activating *PIK3CA*/*PIK3CB* mutations determine p110 isoform dependency in prostate cancer

(A) Growth assay of LNCaP-p110α^{E545K} cells treated with AZD8186 (250nM), AZD8186 (1uM) or vehicle. Cell number was read using CellTiter-Glo assay on Day 1, Day 4, and Day 7, respectively. (B) Growth assay of CWR22Pc-EV cells treated with BYL719 (1μM), AZD8186 (250nM), or Veh (vehicle). Cell number was read using CellTiter-Glo assay on Day 1, Day 4, and Day 7, respectively. (C) Growth assay of CWR22Pc-p110β^{E1051K} cells treated with BYL719 (1μM), AZD8186 (250nM), or vehicle. Cell number was read using CellTiter-Glo assay on Day 1, Day 4 and Day 7, respectively. All assays were performed with three biological replicates. ****$p<0.0001$, A, B, C: one-way ANOVA compared to Veh group, error bar represents mean values ±SD. Source data are provided as Source Data file.

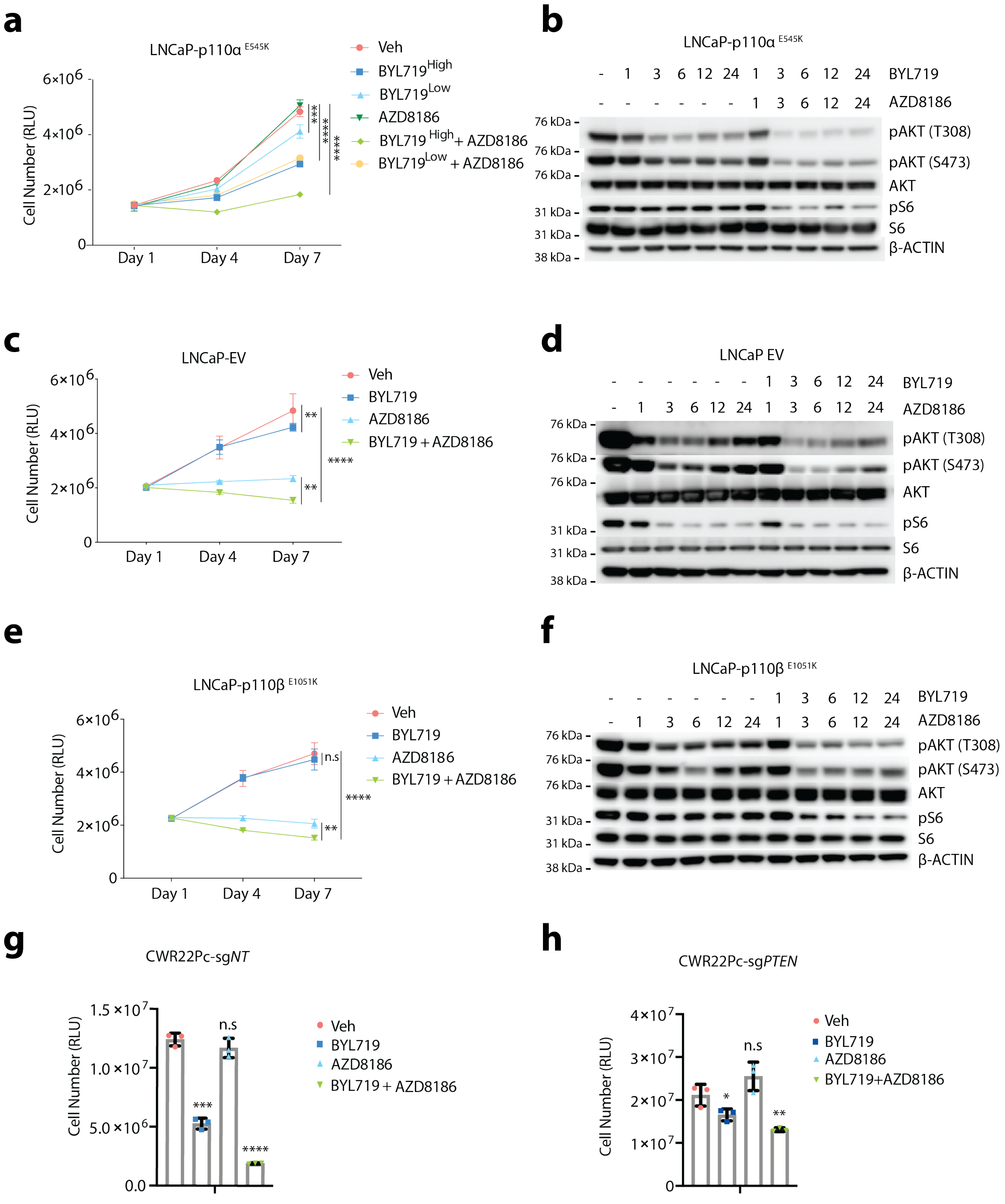

a
LNCaP-p110α E545K
Cell Number (RLU)
6×10^6
4×10^6
2×10^6
0
Day 1
Day 4
Day 7
***
****
****
Veh
BYL719High
BYL719Low
AZD8186
BYL719 High + AZD8186
BYL719Low + AZD8186
b
LNCaP-p110α E545K
- 1 3 6 12 24 1 3 6 12 24 BYL719
- - - - - - 1 3 6 12 24 AZD8186
76 kDa
76 kDa
76 kDa
31 kDa
31 kDa
38 kDa
pAKT (T308)
pAKT (S473)
AKT
pS6
S6
β-ACTIN
c
LNCaP-EV
Cell Number (RLU)
6×10^6
4×10^6
2×10^6
0
Day 1
Day 4
Day 7
**
****
**
Veh
BYL719
AZD8186
BYL719 + AZD8186
d
LNCaP EV
- - - - - - - 1 3 6 12 24 BYL719
- 1 3 6 12 24 1 3 6 12 24 AZD8186
76 kDa
76 kDa
76 kDa
31 kDa
31 kDa
38 kDa
pAKT (T308)
pAKT (S473)
AKT
pS6
S6
β-ACTIN
e
LNCaP-p110β E1051K
Cell Number (RLU)
6×10^6
4×10^6
2×10^6
0
Day 1
Day 4
Day 7
n.s
****
**
Veh
BYL719
AZD8186
BYL719 + AZD8186
f
LNCaP-p110β E1051K
- - - - - - 1 3 6 12 24 BYL719
- 1 3 6 12 24 1 3 6 12 24 AZD8186
76 kDa
76 kDa
76 kDa
31 kDa
31 kDa
38 kDa
pAKT (T308)
pAKT (S473)
AKT
pS6
S6
β-ACTIN
g
CWR22Pc-sgNT
Cell Number (RLU)
1.5 ×10^7
1.0 ×10^7
5.0 ×10^6
0.0
***
n.s
****
Veh
BYL719
AZD8186
BYL719 + AZD8186
h
CWR22Pc-sgPTEN
Cell Number (RLU)
4×10^7
3×10^7
2×10^7
1×10^7
0
*
n.s
**
Veh
BYL719
AZD8186
BYL719+AZD8186

Fig. S3 *PTEN*-deficiency enhances relief of feedback inhibition through p110α and p110β signaling following contralateral isoform selective inhibition

(A) CellTiter-Glo assay showing cell viability of LNCaP-p110α^{E545K} treated with BYL719^{low} (1μM), BYL719^{high} (5μM), AZD8186 (250nM), BYL719^{low} + AZD8186, BYL719^{high} + AZD8186, or vehicle on Day 1, Day 4, and Day 7, respectively Veh: BYL719^{low} Day7, p-value=0.0032. (B) Western blot showing levels of pAKT and pS6 in LNCaP-p110α^{E545K} cells treated with BYL719(5μM), AZD8186 (250nM), BYL719 + AZD8186, or Veh for 0, 1, 3, 6, 12, and 24 hrs, respectively. (C) CellTiter-Glo assay showing cell viability of LNCaP-EV treated with BYL719 (1μM), AZD8186 (250nM), BYL719 + AZD8186, or Veh on Day 1, Day 4, and Day 7, respectively (Day 7 Veh vs. BYL719 p-value=0.0057, AZD8186 vs. BYL719+AZD8186 p-value=0.0016). (D) Western blot showing levels of pAKT and pS6 in LNCaP-EV cells treated with AZD8186 (250nM), BYL719 (1μM) + AZD8186, or vehicle for 0, 1, 3, 6, 12, and 24 hrs, respectively. (E) CellTiter-Glo assay showing cell viability of LNCaP-p110β^{E1051K} treated with BYL719 (1μM), AZD8186 (250nM), BYL719 + AZD8186, or vehicle on Day 1, Day 4, and Day 7, respectively (Day 7 AZD8186 vs. BYL719+AZD8186 p-value=0.0035). (F) Western blot showing levels of pAKT and pS6 LNCaP-p110β^{E1051K} cells treated with AZD8186 (250nM), BYL719 (1uM) + AZD8186, or vehicle for 0, 1, 3, 6, 12, and 24 hrs, respectively. (G, H) CellTiter-Glo assay showing cell viability of CWR22Pc-sg*NT* or CWR22Pc-sg*PTEN* isogenic pairs treated with BYL719 (1μM), AZD8186 (250nM), BYL719 + AZD8186, or Veh, Day 7. G: Veh vs. BYL719 p-value=0.0026; H: Veh vs

BYL719 p-value= 0.004, Veh vs. BYL719+AZD8186 p-value=0.0055. All assays were performed with three biological replicates. ****p<0.0001, ***p<0.001, **p<0.01, **p<0.05, n.s: not significant, A, C, E, G and H: one-way ANOVA compared to Veh group, error bar represents mean values ±SD. Source data are provided as Source Data file.

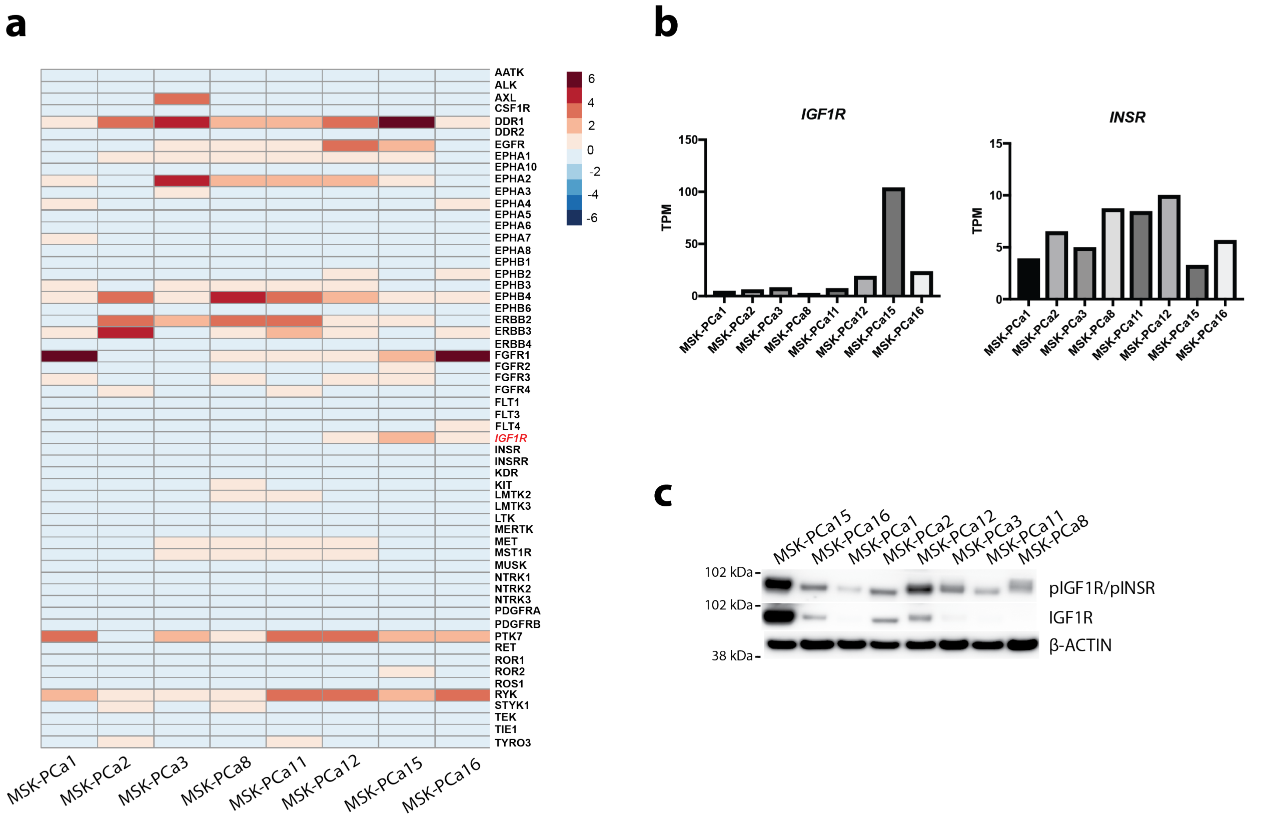

Fig. S4 Differential expression of RTKs across patient derived prostate cancer organoids

(A) Heat map showing RTK transcriptomic analysis across 8 organoid models. (B) Left: Bar graph showing *IGF1R* (left) and *INSR* (right) expression across 8 organoid models. (C) Western blot showing levels of pIGF1R/INSR and IGF1R in a panel of PDO lines. All assays were performed with three biological replicates. Source data are provided as Source Data file.

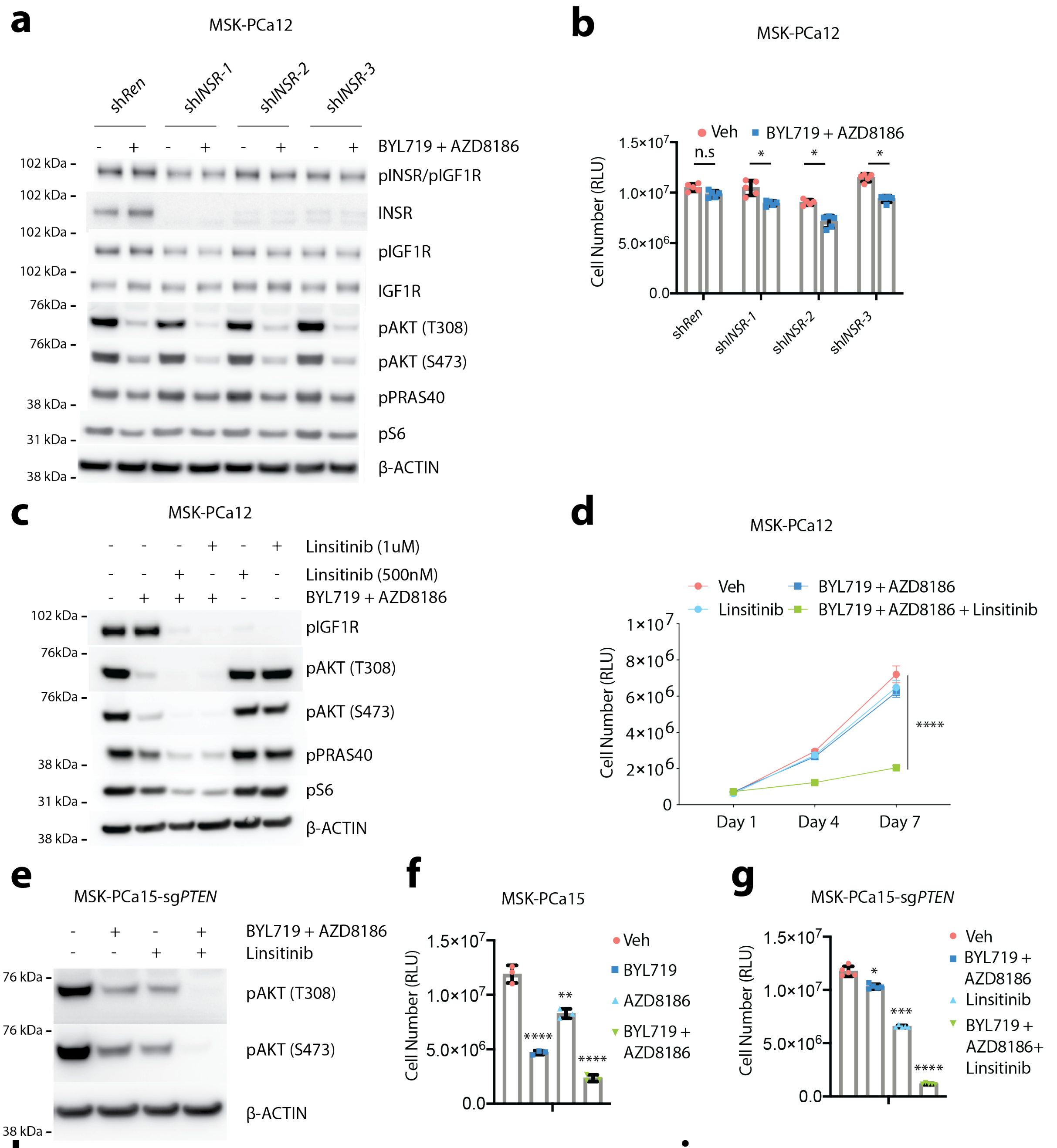

h Number of dead mice bearing MSK-PCa12 xenograft

Time	BYL719+AZD8186	Linsitinib	BYL719+AZD8186 +Linsitinib
Day 0	0	0	0
Day 1	0	0	1
Day 2	0	0	1
Day 3	0	0	1
Day 4	0	0	5

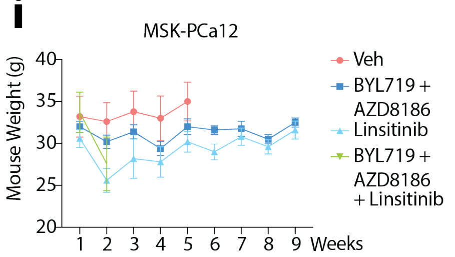

Fig. S5 Upregulation of IGF1R mediates resistance to p110α + p110β blockade

(A) Western blot showing levels of IGF1R/INSR and AKT signaling in MSK-PCa12-sh*Renilla* or MSK-PCa12-sh*INSR* lines treated with BYL719 (1μM) + AZD8186 (250nM), or vehicle for 4 hrs. (B) CellTiter-Glo assay showing cell viability of MSK-PCa12-sh*Renilla* or MSK-PCa12-sh*INSR* organoids treated with BYL719 (1μM) + AZD8186 (250nM) or vehicle, Day 7. shRen p-value=0.04, sh*INSR*-1 p-value=0.02, sh*INSR*-2 p-value=0.02, sh*INSR*-3 p-value=0.01. (C) Western blot showing levels of IGF1R and AKT signaling in MSK-PCa12 organoids treated with linsitinib (500nM, 1μM), BYL719 (1μM) + AZD8186 (250nM), triple combination, or Veh for 4 hrs. (D) Growth assay of MSK-PCa12 organoids treated with linsitinib (1μM), BYL719 (1μM) + AZD8186 (250nM), triple combination, or vehicle. Cell number was read using CellTiter-Glo assay on Day 1, Day 4, and Day 7, respectively. (E) Western blot showing levels of pAKT in MSK-PCa15-sg*PTEN* organoids treated with linsitinib (1μM), BYL719 (1μM) + AZD8186 (250nM), triple combination, or vehicle for 4hrs. (F, G) CellTiter-Glo assay showing cell viability of MSK-PCa15 and MSK-PCa15-sg*PTEN* organoids treated with linsitinib (1μM), BYL719 (1μM) + AZD8186 (250nM), triple combination, or vehicle, Day7. F: Veh vs. AZD8186 p-value=0.0011. G: Veh vs. BYL719+AZD8186 p-value=0.0022, Veh vs. linsitinib p-value=0.0011. (H) Table showing number of dead mice when linsitinib (40mg/kg), BYL719 (25mg/kg) and AZD8186 (75mg/kg), and triple combination were dosed for 4 days. (I) Body weight analysis of mice weight loss with Veh, linsitinib (40mg/kg), BYL719 (25mg/kg) and AZD8186 (75mg/kg), and triple combination (n=5 mice). Source data are provided as Source Data file. All assays were performed with

three biological replicates. ****p<0.0001, ***p<0.001, ***p<0.01, *p<0.05, n.s: not significant, B, D, F and G: one-way ANOVA compared to Veh group, error bar represents mean values ±SD

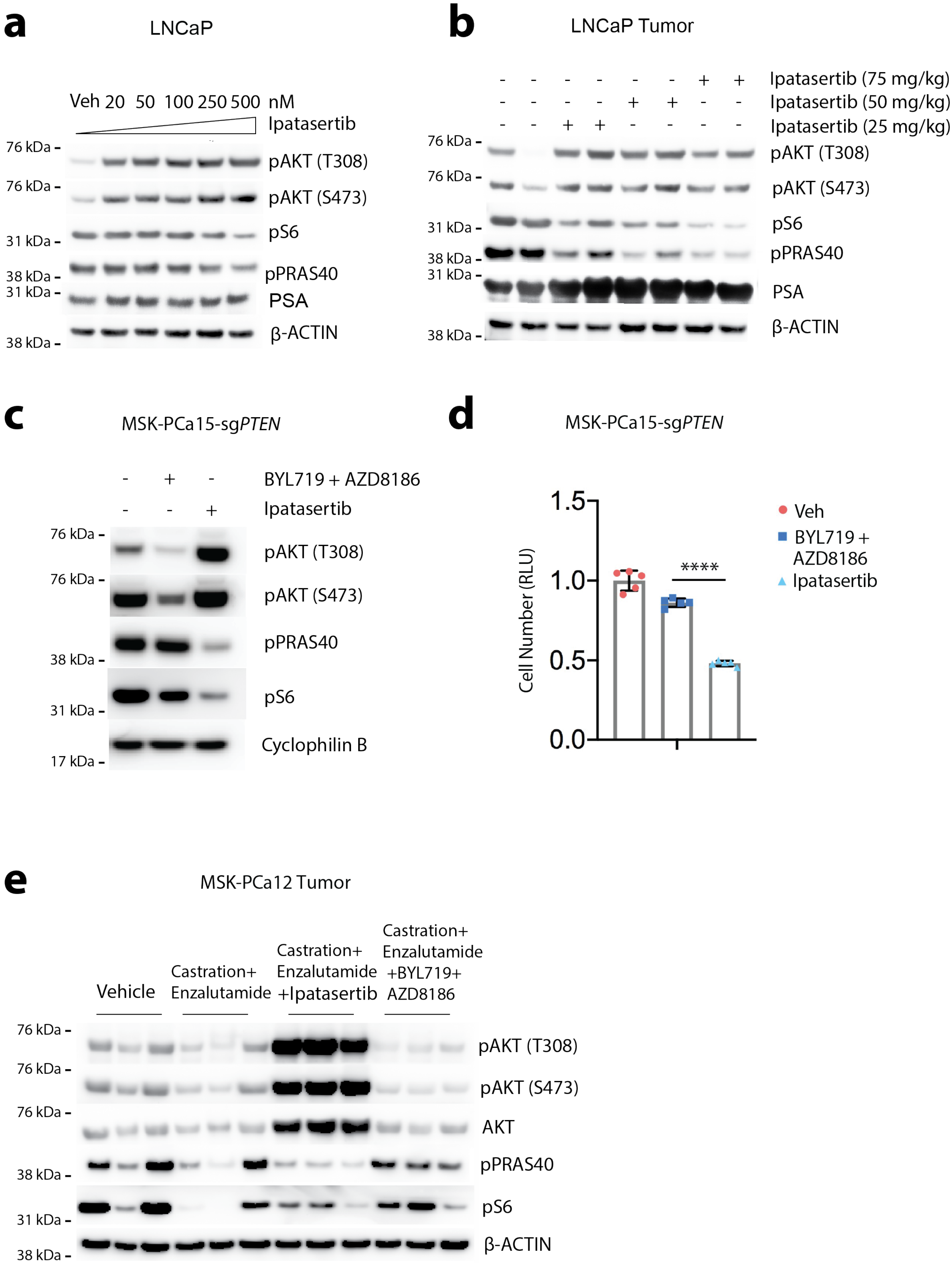
a
LNCaP
Veh 20 50 100 250 500 nM
Ipatasertib
76 kDa
76 kDa
31 kDa
38 kDa
31 kDa
38 kDa
pAKT (T308)
pAKT (S473)
pS6
pPRAS40
PSA
β-ACTIN
b
LNCaP Tumor
Ipatasertib (75 mg/kg)
Ipatasertib (50 mg/kg)
Ipatasertib (25 mg/kg)
pAKT (T308)
pAKT (S473)
pS6
pPRAS40
PSA
β-ACTIN
c
MSK-PCa15-sg*PTEN*
BYL719 + AZD8186
Ipatasertib
pAKT (T308)
pAKT (S473)
pPRAS40
pS6
Cyclophilin B
17 kDa
d
MSK-PCa15-sg*PTEN*
Cell Number (RLU)
1.5
1.0
0.5
0.0
****
Veh
BYL719 + AZD8186
Ipatasertib
e
MSK-PCa12 Tumor
Vehicle
Castration+ Enzalutamide
Castration+ Enzalutamide +Ipatasertib
Castration+ Enzalutamide +BYL719+ AZD8186
pAKT (T308)
pAKT (S473)
AKT
pPRAS40
pS6
β-ACTIN

Fig. S6. AKT-selective inhibition overcomes resistance caused by RTK upregulation

(A) Western blot showing levels of AKT signaling in LNCaP cells treated with escalated dose of ipatasertib (20, 50, 100, 250, and 500nM) or vehicle for 4hrs. (B) Western blot showing levels of AKT signaling in LNCaP tumors treated with escalated dose of ipatasertib (25, 50 and 75mg/kg) or vehicle for 3 days. (C) Western blot showing levels of AKT signaling in MSK-PCa15-sg*PTEN* organoids treated with ipatasertib (500nM) or BYL719 (1μM) + AZD8186 (250nM) for 4 hrs. (D) CellTiter-Glo assay showing cell viability of MSK-PCa15-sg*PTEN* organoids treated with ipatasertib (500nM), BYL719 (1μM) + AZD8186 (250nM) or vehicle, Day7. (E) Western blot showing levels of AKT signaling in MSK-PCa12 tumors from Figure 5C. Source data are provided as Source Data file. All assays were performed with three biological replicates. ****$p<0.0001$, D: one-way ANOVA compared to BYL719+AZD8186 group, error bar represents mean values ±SD
