# Supplementary material for: Defining the therapeutic selective dependencies for distinct subtypes of PI3K pathway-altered prostate cancers
Source: Nat Commun. 2021 Aug 20;12:5053. doi: 10.1038/s41467-021-25341-9 (PMC8379232; doi:10.1038/s41467-021-25341-9)
Supplement: Supplementary file 3 — Description of Additional Supplementary Files [file 41467_2021_25341_MOESM3_ESM.docx]

**Description of Additional Supplementary Files**

Title: Supplementary Data 1

Description: RTK expression data related to supplementary figure S4

Title: Supplementary Data 2

Description: Primer information

Title: Supplementary Data 3

Description: Gene expression profiling of PDOs by RNA-seq (TPM)
