# Supplementary material for: Defining the therapeutic selective dependencies for distinct subtypes of PI3K pathway-altered prostate cancers
Source: Nat Commun. 2021 Aug 20;12:5053. doi: 10.1038/s41467-021-25341-9 (PMC8379232; doi:10.1038/s41467-021-25341-9)
Supplement: Supplementary file 7 — Reporting summary [file 41467_2021_25341_MOESM7_ESM.pdf]

nature research

Corresponding author(s): Brett Carver

Last updated by author(s): Jun 11, 2021

Reporting Summary

Nature Research wishes to improve the reproducibility of the work that we publish. This form provides structure for consistency and transparency in reporting. For further information on Nature Research policies, see our Editorial Policies and the Editorial Policy Checklist.

Statistics

For all statistical analyses, confirm that the following items are present in the figure legend, table legend, main text, or Methods section.

n/a	Confirmed	
[ ]	[x]	The exact sample size (n) for each experimental group/condition, given as a discrete number and unit of measurement
[ ]	[x]	A statement on whether measurements were taken from distinct samples or whether the same sample was measured repeatedly
[ ]	[x]	The statistical test(s) used AND whether they are one- or two-sided<br>*Only common tests should be described solely by name; describe more complex techniques in the Methods section.*
[ ]	[x]	A description of all covariates tested
[ ]	[x]	A description of any assumptions or corrections, such as tests of normality and adjustment for multiple comparisons
[ ]	[x]	A full description of the statistical parameters including central tendency (e.g. means) or other basic estimates (e.g. regression coefficient) AND variation (e.g. standard deviation) or associated estimates of uncertainty (e.g. confidence intervals)
[ ]	[x]	For null hypothesis testing, the test statistic (e.g. F, t, r) with confidence intervals, effect sizes, degrees of freedom and P value noted<br>*Give P values as exact values whenever suitable.*
[x]	[ ]	For Bayesian analysis, information on the choice of priors and Markov chain Monte Carlo settings
[x]	[ ]	For hierarchical and complex designs, identification of the appropriate level for tests and full reporting of outcomes
[x]	[ ]	Estimates of effect sizes (e.g. Cohen's d, Pearson's r), indicating how they were calculated

Our web collection on statistics for biologists contains articles on many of the points above.

Software and code

Policy information about availability of computer code

Data collection	Tumor measuring system Peira TM900
Data analysis	GraphPad Prism (v7 and v8), ImageJ, STAR(v2.3), Cufflinks(v2.1), RStudio(v1.0.143), pheatmap(v1.0.12), RColorBrewer(1.1.2);Samtools(v1.3); Featurecount(v1.4.6),HISAT(v2.0.1),Sambamba(v0.6.6),DEseq2(v.1.4.6)

For manuscripts utilizing custom algorithms or software that are central to the research but not yet described in published literature, software must be made available to editors and reviewers. We strongly encourage code deposition in a community repository (e.g. GitHub). See the Nature Research guidelines for submitting code & software for further information.

Data

Policy information about availability of data

All manuscripts must include a data availability statement. This statement should provide the following information, where applicable:

- Accession codes, unique identifiers, or web links for publicly available datasets
- A list of figures that have associated raw data
- A description of any restrictions on data availability

1. Raw data for figure 1F, 1G and 1H were provided in Raw figure 1.
2. Raw data for figure 2A, 2B, 2C, 2D, 2F, and 2H were provided in Raw figure 2.
3. Raw data for figure 3A, 3C, 3E, 3F and 3H were provided in Raw figure 3.
4. Raw data for figure 4C, 4D, 4G, 5A and 5C were provided in Raw figure 4.
5. Raw data for figure S1A, S1B, S1C, S3B, S3D and S3F were provided in Raw figure S1.
6. Raw data for figure S4B, S5A, S5C, S5E, S6A, S6B, S6C and S6E were provided in Raw figure S2.
7. Raw data for figure 1A, 2E, 2G, 3B, 3D, 3G, 4A, 4B, 4E, 4F, 4H, 4I, 5F, 5G, 5H, S2A, S2B, S2C, S3A, S3C, S3E, S3G, S3H, S5B, S5D, S5F, S5G, S5I and S6D were

provided in file for raw growth assay data
8. Raw data for Figure Raw data from Figure S4 (heatmap) was provided as supplementary table

Field-specific reporting

Please select the one below that is the best fit for your research. If you are not sure, read the appropriate sections before making your selection.

☒ Life sciences ☐ Behavioural & social sciences ☐ Ecological, evolutionary & environmental sciences

For a reference copy of the document with all sections, see nature.com/documents/nr-reporting-summary-flat.pdf

Life sciences study design

All studies must disclose on these points even when the disclosure is negative.

Sample size	No statistical methods were used to pre-determine sample size. Sample sizes were estimated based on pilot experiments, with an effort to achieve a minimum n=3, mostly n=5 mice or replicates per treatment group, which is proved to be sufficient to determine reproducible results.
Data exclusions	No data were excluded from analyses
Replication	All experiments were reliably reproduced. All immunoblots were performed with three independent biological repeats. Representative and consistent findings were shown. Both in vitro and in vivo grow assays were repeated at least with two independent biological repeats, all attempts at replication were successful.
Randomization	For in vivo studies, aged-matched mice bearing tumors were randomized by average tumor size in each groups. Only male mice were used since prostate cancer only occurs in men. For in vitro studies, samples were randomized before starting treatment.
Blinding	For in vivo studies, investigators were not directly involved in raw data collection. Dosing and tumor size measurement was performed at anti-tumor assessment core facility. Therefore, the data collection was blinded to investigators. For in vitro studies, analysis was objective and did not require blinding.

Reporting for specific materials, systems and methods

We require information from authors about some types of materials, experimental systems and methods used in many studies. Here, indicate whether each material, system or method listed is relevant to your study. If you are not sure if a list item applies to your research, read the appropriate section before selecting a response.

Materials & experimental systems

n/a	Involved in the study	
☐	☒	Antibodies
☐	☒	Eukaryotic cell lines
☒	☐	Palaeontology and archaeology
☐	☒	Animals and other organisms
☒	☐	Human research participants
☒	☐	Clinical data
☒	☐	Dual use research of concern

Methods

n/a	Involved in the study	
☒	☐	ChIP-seq
☒	☐	Flow cytometry
☒	☐	MRI-based neuroimaging

Antibodies

Antibodies used	AR (Abcam, ab108341, Lot: GR3186826-6),β-actin(13E5) (Cell Signaling Technology, 4970S, Lot: 15), phospho-AKT (Ser473)(D9E) (Cell Signaling Technology, 4060L, Lot: 25), phospho-AKT (Thr308)(D25E6) (Cell Signaling Technology, 4056S, Lot: 7), AKT(11E7) (Cell Signaling Technology, 4685S, Lot: 6), phospho-S6 ribosomal protein (S235/236)(2F9)(Cell Signaling Technology, 4856S, Lot: 9), S6 (5G10) ribosomal protein (Cell Signaling Technology, 2217S, Lot: 7), PTEN(D4.3) (Cell Signaling Technology, 9188L, Lot: 6), phospho-PRAS40 (Thr246)(C77D7)(Cell Signaling Technology, 2997S, Lot: 12), PRAS40(D23C7) (Cell Signaling Technology, 2691S, Lot: 8), phospho-IGF1R (Tyr1135/1136)/INSR (Tyr1150/1151)(19H7) (Cell Signaling Technology, 3024S, Lot: 15), phospho-IGF1R (Tyr1135) (DA7A8) (Cell Signaling Technology, 3918S, Lot: 2), IGF1R(D23H3) (Cell Signaling Technology, 9750S, Lot: 5), INSR(4B8) (Cell Signaling Technology, 3025S, Lot: 19), phospho-p44/42 MAPK (Erk1/2) (Thr202/Tyr204) (D13.14.4E) XP (Cell Signaling Technology, 4370S, Lot: 17), p44/42 MAPK (Erk1/2) (137F5) (Cell Signaling Technology, 4695S, Lot: 21), phospho- MEK1/2 (Ser217/221) (41G9) (Cell Signaling Technology, 9154S, Lot: 18), MEK1/2 (D1A5) (Cell Signaling Technology, 8727S, Lot: 5), PI3K kinase P110α(C73F8) (Cell Signaling Technology, 4249S, Lot: 10), PI3K kinase P110β(C33D4) (Cell Signaling Technology, 3011S, Lot: 6), HSP90(E289) (Cell Signaling Technology, 4875S, Lot: 3), Cyclophilin B(D1V5J) (Cell Signaling Technology, 43603S, Lot: 1), DDK tag(Clone OTI4C5) (Origene, TA50011-100, Lot: W031).
Validation	AR (Abcam, ab108341): Human, WB, Citations(29)

β-actin(13E5) (Cell Signaling Technology, 4970S): Human, WB, Citations(2918)
phospho-AKT (Ser473)(D9E) (Cell Signaling Technology, 4060L): Human, WB, Citations(5677)
phospho-AKT (Thr308)(D25E6) (Cell Signaling Technology, 4056S): Human, WB, Citations(542)
AKT(11E7) (Cell Signaling Technology, 4685S): Human, WB, Citations(1094)
phospho-S6 ribosomal protein (S235/236)(2F9)(Cell Signaling Technology, 4856S): Human, WB, Citations(215)
S6 (5G10) ribosomal protein (Cell Signaling Technology, 2217S): Human, WB, Citations(1336)
PTEN(D4.3) (Cell Signaling Technology, 9188L): Human, WB, Citations(396)
phospho-PRAS40 (Thr246)(C77D7)(Cell Signaling Technology, 2997S): Human, WB, Citations(149)
PRAS40(D23C7) (Cell Signaling Technology, 2691S): Human, WB, Citations(118)
phospho-IGF1R (Tyr1135/1136)/INSR (Tyr1150/1151)(19H7) (Cell Signaling Technology, 3024S): Human, WB, Citations(247)
phospho-IGF1R (Tyr1135)(DA7A8) (Cell Signaling Technology, 3918S): Human, WB, Citations(50)
IGF1R(D23H3) (Cell Signaling Technology, 9750S): Human, WB, Citations(141)
INSR(4B8) (Cell Signaling Technology, 3025S): Human, WB, Citations(208)
phospho-p44/42 MAPK (Erk1/2) (Thr202/Tyr204) (D13.14.4E) XP (Cell Signaling Technology, 4370S): Human, WB, Citations(4677)
p44/42 MAPK (Erk1/2) (137F5) (Cell Signaling Technology, 4695S): Human, WB, Citations(3531)
phospho- MEK1/2 (Ser217/221) (41G9) (Cell Signaling Technology, 9154S): Human, WB, Citations(523)
MEK1/2 (D1A5) (Cell Signaling Technology, 8727S): Human, WB, Citations(120)
PI3K kinase P110α(C73F8) (Cell Signaling Technology, 4249S): Human, WB, Citations(367)
PI3K kinase P110β(C33D4) (Cell Signaling Technology, 3011S): Human, WB, Citations(68)
HSP90(E289) (Cell Signaling Technology, 4875S): Human, WB, Citations(35)
Cyclophilin B(D1V5J) (Cell Signaling Technology, 43603S): Human, WB, Citations(8)
DDK tag(Clone OTI4C5m Host: Mouse) (Origene, TA50011-100): WB, Citations(204) (PMID: 31451622, 30181214, 6187133)
For all antibodies used, please refer to manufacturer's web site.

Eukaryotic cell lines

Policy information about cell lines

Cell line source(s)

Patient-derived organoids (PDOs) (MSK-PCa1, MSK-PCa2, MSK-PCa3, MSK-PCa8, MSK-PCa11, MSK-PCa12, MSK-PCa15, MSK-PCa16, BM110) were generated as previously described(Gao, D. et al., Wang et al., unpublished). Acquisition of human tissue for prostate cancer organoids was performed under MSKCC IRB-approved protocols # 06-107 and 12-001. LNCaP (#ATCC® CRL-1740™) and DU145 (#ATCC® HTB-81™) were obtained from American Type Culture Collection (ATCC, Manassas, VA). MCF7(#ATCC® HTB-22™), T47D(#ATCC® CRL-2865™), MDA-MB-453(#ATCC® HTB-131™), EFM19(DSMZ, #ACC-231), CAL51(DSMZ, #ACC-231) and BT20 (#ATCC® HTB-19™) cell lines were gifts from Dr. Guotai Xu (MSKCC). CWR22Pc was a gift from Marja T. Nevalainen (Thomas Jefferson University, Philadelphia, PA). CWR22Pc is not available through commercial source.

Authentication

Cell lines that were directly purchased from ATCC or DSMZ were authenticated through ATCC STR services. For human organoid lines, STR testing was performed as previous described (Gao et al., Cell 2014). STR testing for CWR22Pc was performed as previously described (Mu et al., Science, 2017).

Mycoplasma contamination

All cell lines and prostate cancer organoids used in our studies have tested negative for mycoplasma using the MycoProbe Mycoplasma Detection Kit (R&D Systems) within one month of initiating experiments.

Commonly misidentified lines
(See ICLAC register)

NO

Animals and other organisms

Policy information about studies involving animals; ARRIVE guidelines recommended for reporting animal research

Laboratory animals

Animals were acclimatized for at least one week prior to study initiation. Mice were housed in polycarbonate cages with micro-isolator tops (5 mice/cage). Absorbent heat-treated hardwood bedding was used with cage changes once per week. Certified Purina Pico Chow No. 5002 was provided ad libitum. The manufacturer's composition formula was included in the raw data and reviewed prior to use. Reverse osmosis (RO) treated tap water was provided ad libitum in polycarbonate water bottles with stainless steel sipper tubes. Water bottles were changed once per week. The ambient temperature was kept between 20.9–23.9°C. A 12/12 hour light/dark cycle was used. Mouse strain: SCID: NOD.Cg-Prkdc<scid> Il2rg<tm1Wjl>/SzJ, Vendor: JACKSON LABORATORY.
Age: 8 weeks, Sex: male.

Wild animals

No wild animals were used in the study.

Field-collected samples

No field collected samples were used in the study.

Ethics oversight	All experiments involving animals were approved by MSKCC Animal Ethics Committee.

Note that full information on the approval of the study protocol must also be provided in the manuscript.
